# Clinical Profile of Adult Bronchial Asthma Patients Presenting at a Tertiary Care Teaching Institute in Northern India

**DOI:** 10.7759/cureus.39316

**Published:** 2023-05-21

**Authors:** Subodh Kumar, Devesh P Singh, Rama S Rath, Garima Kushwaha, Sana Ansari, Dineshwar K Rai, Umesh C Ojha, Aroop Mohanty

**Affiliations:** 1 Pulmonary Medicine, All India Institute of Medical Sciences (AIIMS) Gorakhpur, Gorakhpur, IND; 2 Community Medicine, All India Institute of Medical Sciences (AIIMS) Gorakhpur, Gorakhpur, IND; 3 Pulmonary Medicine, ESI Post Graduate Institute of Medical Sciences and Research, New Delhi, IND; 4 Clinical Microbiology, All India Institute of Medical Sciences (AIIMS) Gorakhpur, Gorakhpur, IND

**Keywords:** gender distribution in asthma, demographic characteristics of asthma, rhinitis, clinical profile of asthma patients, asthma

## Abstract

Background: In the previous four decades there have been remarkable changes and development in the approach toward the diagnosis and management of asthma. There are wide variations in the clinical profile of asthma patients in different parts of a vast country like India due to significant variations in the geography, culture, ethnicity, and socioeconomic profile of the Indian population. In the present study, we have aimed to study the clinical profile of adult asthmatic patients in a tertiary care teaching institute in Northern India.

Methods: In this observational cross-sectional study, a total of 966 asthma patients were included from August 2020 to July 2021 after following strict inclusion and exclusion criteria. After a thorough history and clinical examination, patients were subjected to relevant investigations including spirometry.

Results: Our study showed slight female preponderance (51.7%) over males among asthma patients. A maximum number of patients were of comparatively younger age groups and urban. The most common symptom at the time of presentation was breathlessness (94.5%) followed by cough in about 59.8%. Family history was present in about 9.3% of patients. A maximum number of patients presented in the months of November and December and rhinitis was the most commonly associated atopic condition. The majority (65.28%) of previously diagnosed patients had uncontrolled asthma at the time of their first presentation in our department.

Conclusion: Results of our study endorse the poor awareness in society towards education and management of asthma. Females and comparatively younger patients are more commonly affected. Significant differences in our study from previous studies in different parts of India confirm that the pattern and clinical profile of asthma patients in one region cannot be extrapolated to other regions and the need for future studies in other regions of our country is also required.

## Introduction

Obstructive airway diseases are one of the major causes of morbidity and mortality throughout the world. Bronchial asthma is one of the most common obstructive airway diseases. In the last four decades, there have been remarkable changes and development in the approach toward the diagnosis and management of asthma. Asthma is described as a heterogeneous disease, characterized by chronic airway inflammation which results in respiratory symptoms like breathlessness, wheezing, tightness of the chest, and cough [1]. Previous studies have reported the prevalence of asthma ranging from 2% to 23% among Indian children and adults [2-6]. This huge variation in prevalence may be because of significant variations in geography, culture, ethnicity, and socioeconomic profile of India as well as the difference in study methodologies. Till now, only very few studies have provided data about the clinical profile of asthma patients in India. In the present study, we have aimed to study the clinical profile of adult asthmatic patients in a tertiary care teaching institute in Gorakhpur, UP.

## Materials and methods

This study was an observational cross-sectional study, conducted between August 2020 and July 2021 in the outpatient department of Pulmonary Medicine at All India Institute of Medical Sciences (AIIMS), Gorakhpur, Uttar Pradesh, India. Diagnosis of asthma was established based on the history, clinical presentation, and diagnostic criteria as per Global Initiative for Asthma (GINA) 2020.

All patients presenting with complaints of shortness of breath, cough (with or without expectoration), tightness of chest and wheezing, and fulfilling bronchodilator reversibility criteria on spirometry as per GINA guidelines 2020 or those who are previously diagnosed as a patient of asthma were included in the study.

All patients with diagnosed active pulmonary tuberculosis diagnosed with cardiac failure or left ventricular failure, and myocardial infarction were excluded from the study. A patient with diagnosed COVID-19 either by rapid antigen testing or polymerase chain reaction or with high clinical suspicion of COVID-19 although not microbiologically proven was also excluded from the study. 

Proper history taking and clinical examination were done thoroughly on all the patients. Recordings of systolic and diastolic blood pressure, oxygen saturation, pulse rate, respiratory rate, height, and weight were done in all patients. A complete blood count was done in all patients. Chest X-ray was done in all patients except pregnant patients. Objective assessment of variable expiratory airflow limitation was done with spirometry criteria as per GINA guidelines 2020. After baseline recording of forced expiratory volume in the first second (FEV1), patients were given 200 mcg of salbutamol and after 15 min spirometry was repeated. For objective assessment and confirmation of variable expiratory airflow limitation, the reversibility criteria used in our study was an improvement in FEV1 as 12% (or more) + 200 mL (or more) after giving metered dose inhaler of 200 mcg of salbutamol [1].

The study protocol has been approved by the institutional human ethics committee (IHEC) with IHEC Reference number - IHEC/AIIMS-GKP/BMR/137/2023 dated 25-March-2023.

## Results

**Figure 1:**
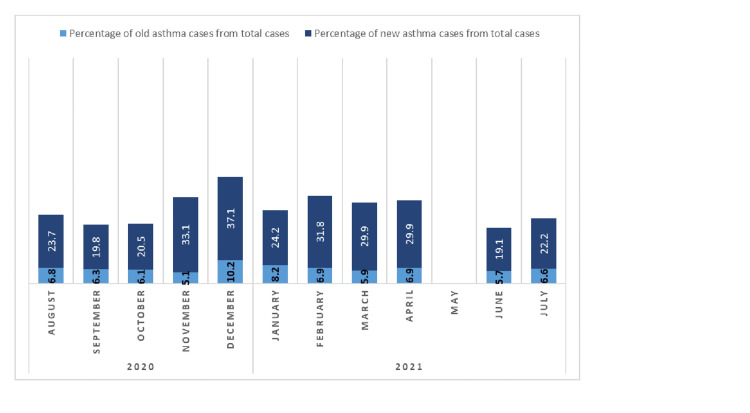
Monthwise distribution of asthma cases.

**Figure 2:**
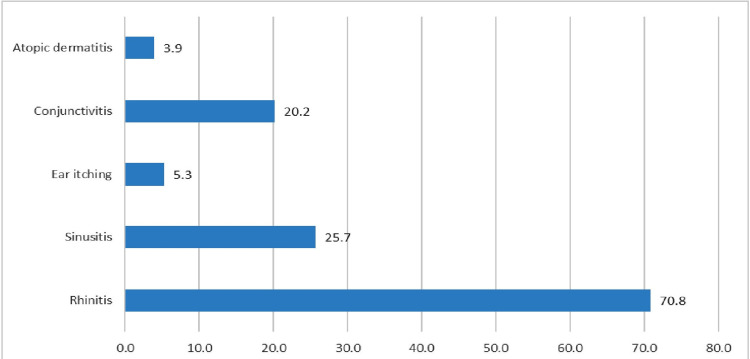
Associated allergic conditions among asthma patients.

A total of 2864 patients reported to the Out-Patient Department of which 1187 were diagnosed as cases of asthma. Of the total 1187 diagnosed cases, 221 were excluded and rest 966 patients were included in our study. Our study showed a slight female preponderance (51.7%) among asthma patients. The highest number of cases were belonging to the age group between 18-30 and 31-45 years of age, together constituting about 63% of patients. The majority of patients were urban dwellers (59.1%) and never smokers in our study (94.72%). Family history was present in about 9.3% patients with asthma (Table 1). About 80% of patients were newly diagnosed at the time of presentation, and the rest 20% were previously diagnosed. The most common symptom at the time of presentation was breathlessness (94.5%) followed by cough in about 59.8% of patients (Table 1). Out of the total of 193 previously diagnosed cases presenting to our outpatient department (OPD), a majority (65.28%) had uncontrolled asthma according to their level of symptom control defined as per GINA guidelines 2020. The trend of the reported cases of asthma over the study period is shown in Figure 1. The majority of the cases were reported in the winter and fall months, i.e. December-March. Very few cases were reported in summer and in the rainy season whereas no cases were seen during the month of May as our institute was under COVID-19 protocol where the OPD services were temporarily closed. Rhinitis followed by sinusitis was the most common allergic (atopic) condition associated with asthma in the study followed by sinusitis (Figure 2).

**Table 1:** Socio-behavioral pattern of patients reported to the OPD. OPD, outpatient department

Socio-behavioral characteristics		N (%)
Gender	Male	466 (48.24%)
	Female	500 (51.76%)
Age	18-30	303 (31.36%)
	31-45	304 (31.46%)
	46-60	206 (21.32%)
	More than 60	153 (15.83%)
Place of residence	Urban	571 (59.1%)
	Rural	395 (40.89%)
Smoking status	Past smoker	34 (3.52%)
	Active smoker	17 (1.76%)
	Non-smoker	915 (94.72%)
Diagnosis	New	773 (80.02%)
	Old	193 (19.98%)
Symptoms	Breathlessness	913 (94.5%)
	Cough	578 (59.8%)
	Chest tightness	195 (20.1%)
	Wheeze	297 (30.7%)
Family history of asthma in first-degree relatives		90 (9.3%)

## Discussion

The current study is a hospital-based study conducted in the northern part of India. We found female preponderance among the asthma patients. Several studies in past have revealed a significantly higher percentage of males as compared to females among asthmatic patients including a study over the adult Nigerian population and another study done in Assam, India but our study has shown slight female preponderance [7-9]. This may be due to the fact that the study is a hospital-based study. Although, the ONEAIR study, done in Spain and published in the year 2008 has shown clear female preponderance [10].

Our study has shown a higher prevalence of asthma among the Urban population which is again similar to the ONEAIR study [10]. The majority of patients in our study fall in the age group between 18 and 45 years which is similar to studies done at Assam in India as well as in Nigeria [8-9]. More than 94% of patients in our study were non-smokers. Similar data were obtained in an Indian study done in Rajasthan [11]. More than 80% of patients were newly diagnosed in our department. The most common symptom in adult asthmatic patients in our study was breathlessness although Globe et al. has reported chest tightness followed by wheezing as the most common symptom in an American study [12]. Most of the other studies have reported cough as the most common symptom [7-8].

Family history of asthma was present in approximately 9% of patients in our study; however, family history was found in about 23% of asthmatics in the Nigerian study [8]. An Indian study by Singh et al. done in Rajasthan reported family history in about 35% of cases [11]. In our study, a maximum number of patients presented in the month of November and December followed by a second peak in the months of February, March, and April. This may be related to significant changes in weather conditions in North India in the above-mentioned months and the presence of pollens in higher concentrations between February and April. Nearly similar observations were seen in the study by Singh et al. [11]. The majority of previously diagnosed asthma patients (about 65%) presenting to our outpatient department had uncontrolled asthma at the time of their presentation. This shows how poorly, asthma patients have been managed in the community as well as the lack of awareness toward control and management of asthma among common people and healthcare providers in the private sector. This data support several other studies published previously [13-17]. Rhinitis followed by sinusitis was the most commonly associated allergic/atopic conditions in our study. Nearly similar observations were found in several other studies in past [18-21].

The study has a few limitations. The study is a hospital-based study thus the prevalence of the disease under study cannot be calculated. Further, no sample size was calculated in this study, which could have led to less power of the study to estimate the clinical profile of the disease.

## Conclusions

We found the majority of asthma cases are not properly diagnosed at the local health system level and remain hidden carrying the burden of morbidity. This may be a subject of future follow-up studies owing to the unique situation in this part of the country. Although the prevalence of the study cannot be extrapolated to the high burden of the cases in the area, due to the institutional nature of the study.
